# The Effect of Nanoscale Modification of Nisin by Different Milk-Derived Proteins on Its Physicochemical Properties and Antibacterial Activity

**DOI:** 10.3390/foods13111606

**Published:** 2024-05-22

**Authors:** Jing Wang, Rui Liu, Xiaoyang Huang, Yuexin Bao, Xiaohong Wang, Huaxi Yi, Youyou Lu

**Affiliations:** 1 College of Food Science and Technology, Huazhong Agricultural University, Wuhan 430070, China; wangjing001128@163.com (J.W.); 13647220876@163.com (R.L.); hxy_mbdtf0818@163.com (X.H.); byx9812276@163.com (Y.B.); wxh@mail.hzau.edu.cn (X.W.); 2 Key Laboratory of Environment Correlative Dietology, Ministry of Education, Huazhong Agricultural University, Wuhan 430070, China; 3 College of Food Science and Engineering, Ocean University of China, Qingdao 266000, China; yihx@ouc.edu.cn

**Keywords:** physicochemical, milk-derived proteins, nisin, antibacterial properties, nanoparticles

## Abstract

Nisin is used as a natural food preservative because of its broad-spectrum antimicrobial activity against Gram-positive bacteria. However, free nisin is susceptible to various factors that reduce its antimicrobial activity. Milk protein, a protein derived from milk, has self-assembly properties and is a good carrier of bioactive substances. In this study, lactoferrin–nisin nanoparticles (L-N), bovine serum albumin–nisin nanoparticles (B-N), and casein–nisin nanoparticles (C-N) were successfully prepared by a self-assembly technique, and then their properties were investigated. The studies revealed that lactoferrin (LF) and nisin formed L-N mainly through hydrophobic interactions and hydrogen bonding, and L-N had the best performance. The small particle size (29.83 ± 2.42 nm), dense reticular structure, and good thermal stability, storage stability, and emulsification of L-N laid a certain foundation for its application in food. Further bacteriostatic studies showed that L-N enhanced the bacteriostatic activity of nisin, with prominent inhibitory properties against *Listeria monocytogenes*, *Staphylococcus aureus*, and *Bacillus cereus*, which mainly disrupted the cell membrane of the bacteria. The above results broaden our understanding of milk protein–nisin nanoparticles, while the excellent antibacterial activity of L-N makes it promising for application as a novel food preservative, which will help to improve the bioavailability of nisin in food systems.

## 1. Introduction

Nisin is a natural antimicrobial peptide produced by *Lactococcus lactis* subsp. *lactis*, which consists of 34 amino acids and has a molecular weight of ~3.35 kDa [1]. Nisin exhibits broad-spectrum antimicrobial activity against Gram-positive bacteria, so it is often used as a natural food preservative [2,3]. Its antimicrobial pathway involves two main types including the formation of pores in the bacterial cell membrane and the interaction with highly conserved cell wall synthesis intermediates [4,5]. The high affinity of nisin for lipid II, an essential precursor in peptidoglycan biosynthesis, causes depolarization of the cell membrane, resulting in the formation of channels in the cell membrane that disrupt the proton motive force and pH balance, leading to ion efflux, ATP depletion, and leakage of cellular contents, which in turn leads to cell death [6]. Meanwhile, peptidoglycan is an important material for cell wall synthesis, and the binding of nisin and lipid II prevents the synthesis of peptidoglycan, thus further disrupting the biosynthesis of the cell wall and its functional role. As an antimicrobial peptide, nisin is generally recognized as safe (GRAS) by the World Health Organization (WHO) and the Food and Drug Administration (FDA) of the U.S. and is currently approved for use in more than 80 countries and regions [7]. Nisin has been widely used in various industries, such as the biomedical, pharmaceutical, agricultural, and food fields. It has been used in a variety of food products, including dairy products, meat products, beverages, and vegetables, which can effectively inhibit the contamination of food-borne pathogens and prolong the shelf life of foods [8]. However, the antimicrobial activity of nisin is reduced in practical applications because of its interaction with food matrices, low resistance to proteases, and poor stability at pH levels close to neutral and alkaline [9,10,11]. For this challenge, nano-encapsulation technology may potentially be an excellent strategy to solve the above problems [12,13,14]. In the study by Niaz, et al. [15], nisin Z was encapsulated into rhamnosome nano-vesicles, which were formed by combining rhamnolipids with nano-liposome membranes, and the nano-vesicles had broad-spectrum antimicrobial activity. Meanwhile, the efficiency of encapsulating nisin increased from 47% to 88% after doping rhamnolipids into the lipid bilayer.

Among the range of nano-encapsulated materials, proteins have been widely noticed and used for their rich nutritional value, unique functional properties, and versatility in altering their structure [16]. Compared with other sources of proteins, milk proteins (specifically referred to as bovine milk proteins in this study) show more potential as nanocarriers for bioactive substances because milk proteins, as high-quality proteins containing essential and non-essential amino acids required by the human body with a high nutritional value, are more easily digested by the human body and have become an integral part of the daily human diet, and they are usually considered as GRAS [17]. Milk protein mainly includes the following two categories: whey protein and caseins (CNs). Whey protein accounts for about 20%, and CNs account for about 80%. Among them, whey protein is mainly composed of β-lactoglobulin and α-lactalbumin, as well as functional proteins such as lactoferrin (LF), bovine serum albumin (BSA), and immunoglobulin, the content of which is relatively low and is mostly processed wastes from cheese [18,19]. LF is a non-heme iron-binding protein belonging to the transferrin family with a molecular weight of ~80 kDa and an isoelectric point (pI) of 8. It possesses a variety of functions, including antibacterial, antifungal, antiviral, antiparasitic, and antioxidant, and it is the most extensively studied of the whey proteins, which binds to a wide range of compounds, such as DNA, proteins, polysaccharides, and metal ions [20,21]. BSA is a giant globular protein with a molecular weight of about 66 kDa and a pI of 4.7. It has a great homology with human serum albumin and can bind extensively to various substances that are of significant physiological and clinical importance [22]. CNs are phosphoproteins belonging to the family of phosphoproteins with the following four types: α_S1_-CN, α_S2_-CN, β-CN, and κ-CN, with a molecular weight of ~19–25 kDa and pI of 4.6. CNs contain a large amount of proline, and they are considered unstructured or naturally denatured proteins with an open structure that can easily be modified [23].

Protein-based nanocarriers have a variety of structural forms, such as nanoparticles, nanoemulsions, nanoliposomes, nanotubes, and nanofibers, among which nanoparticles have attracted much attention because of their unique properties, such as their tunable nanosize and large specific surface area and adaptable chemical, physical, and mechanical properties, which provide distinct advantages for the delivery of bioactive substances [24,25]. The structure of nanoparticles consists of a dense polymer network in which bioactive substances can be dispersed [26]. Self-assembly is one of the critical methods for the formation of nanoparticles from proteins. The self-assembly of proteins is the process of the spontaneous organization of macromolecules from a disordered state into a highly ordered state. These ordered structures are in thermodynamic equilibrium, which is influenced by environmental factors such as pH, temperature, and pressure [27]. Protein self-assembly can be induced by the reversible or irreversible aggregation of protein fragments driven by covalent or non-covalent interactions, such as hydrogen bonding, van der Waals forces, π-π stacking, and host–guest and hydrophobic interactions [28]. Self-assembly also has many advantages including the following: firstly, it is accurate and reproducible and requires little energy; secondly, it can enhance the stability of bioactive substances and prevent degradation; thirdly, when environmental conditions change, the formation of supramolecular structures can be triggered or reversed [29]. LF, BSA, and CNs in milk have self-assembling properties. Yang, Liang, et al. [30] successfully developed ternary complexes consisting of LF, oat β-glucan (OG), and curcumin (Cur) with three binding sequences by using self-assembly and spray-drying techniques, which could be used as potential emulsifiers for stabilized oil-in-water Pickering emulsions. The order of the emulsifying ability of the complexes was as follows: spray-dried ternary complex > spray-dried LF-OG complex > self-assembled ternary complex > self-assembled LF-OG complex. On the other hand, some researchers [31] investigated thermally induced BSA aggregation on a molecular scale using spectroscopic techniques in the temperature range of 12–84 °C and at pH 5.0. Ouyang et al. [32] prepared modified casein nanoparticles (MCs) for the encapsulation of cyanidin 3-O-glucoside (C3G) at pH 5.5 after heating at 80 °C for 30 min and found that the two were bound mainly through hydrophobic interactions. The addition of CN significantly prevented the thermal degradation (90 °C/30 min), oxidation (15% H_2_O_2_/1 h), photodegradation (30 h), and storage stability of C3G.

There is currently no in-depth report on how to comprehensively reveal the effects and mechanisms of different milk proteins on the stability and antibacterial activity of nisin, especially milk protein–nisin nanoparticles prepared by a self-assembling process. This is an urgent and important problem to be solved at present.

In this study, different milk proteins (LF, BSA, CN) were used as nanocarriers to encapsulate nisin via their self-assembly properties and prepare different milk protein–nisin nanoparticles. The structures and properties of three milk protein–nisin nanoparticles were characterized by various means, such as scanning electron microscopy (SEM), Fourier transform infrared (FTIR) spectroscopy, ultraviolet–visible (UV–vis) spectroscopy, and fluorescence spectroscopy, and their antibacterial activity was analyzed. The method had the advantages of simple operation and flexible preparation, which expanded the application range of nisin, improved the utilization of milk proteins, and provided a theoretical basis and a new method for the development of novel antibacterial materials.

## 2. Materials and Methods

### 2.1. Materials

LF was purchased from Beston Pure Dairies Pty Ltd. (Wayville, Australia). BSA, CN, nisin, and dithiothreitol (DTT) were purchased from Solarbio Co., Ltd. (Beijing, China). Jinlongyu soybean oil was purchased from a local supermarket (Wuhan, China). Luria–Bertani nutrient broth medium and Luria–Bertani nutrient agar medium were purchased from Qingdao Gaokoyuan Haibo Biotechnology Co., Ltd. (Qingdao, China). All other chemicals used were purchased from Sinopharm Chemical Reagent Co., Ltd. (Shanghai, China).

*Listeria monocytogenes* (*L. monocytogenes* ATCC19114), *Staphylococcus aureus* (*S. aureus* ATCC25923), *Bacillus cereus* (*B. cereus* CMCC63303), and *Pseudomonas fluorescens* (*P. fluorescens* ATCC13525) were preserved strains in our laboratory.

### 2.2. Preparation of Different Milk Protein–Nisin Nanoparticles

The different milk protein–nisin nanoparticles were prepared according to the optimal conditions obtained from previous experiments. Briefly, LF (4 mg/mL), BSA (3 mg/mL), and CN (5 mg/mL) were dissolved in distilled water, and nisin (5 mg/mL) was dissolved in a 0.02 mol/L HCl solution. The solution was placed on a magnetic stirrer for 2 h and left at 4 °C overnight before being filtered through a 0.22 μm filter. The nisin solution was added drop by drop to the milk protein solution (milk protein: nisin = 1:1, *v*/*v*) with stirring for 2 h. Subsequently, the pH was adjusted to pH 5.0 for LF–nisin (L-N) and BSA–nisin (B-N), and pH 4.0 for CN–nisin (C-N) and then heated in a water bath for 30 min at 50 °C, 40 °C, and 50 °C, respectively, followed by an immediate ice bath, and left overnight at 4 °C to form a transparent or opaque composite. The overnight composite was further processed with an ultrasonic cell crusher (VCX750, Sonics, Newtown, CT, USA, USA) with ultrasonic power of 240 W, 270 W, and 270 W and ultrasonic time of 5 min, 15 min, and 15 min, respectively. Finally, the complex solution was freeze-dried (Beta 2–8 LD, Christ, Osterode, Germany) to obtain milk protein–nisin nanoparticles, which were stored at 4 °C for further use.

### 2.3. Characterization of Different Milk Protein–Nisin Nanoparticles

Multiple experimental methods were used to characterize the various properties of different milk protein–nisin nanoparticles, such as particle size, the polydispersity index (PDI), zeta potential, SEM (S470, Hitachi, Tokyo, Japan), FTIR (Vertex 70, Bruker, Germany), UV–vis spectrum (UV-1800, Shimadzu, Japan), fluorescence spectrum (F-4600, Hitachi, Tokyo, Japan), circular dichroism (CD) spectrum (J-1500, JASCO, Tokyo, Japan), X-ray diffraction (XRD, D8 Advance, Rigaku, Osaka, Japan), and differential scanning calorimetry (DSC,204F1, Netzsch, Selb, Germany). The detailed parameters of each characterization can be found in the Supporting Information.

### 2.4. Determination of Intermolecular Interaction Forces

The molecular interaction forces between nanoparticles were determined by measuring the change in particle size of nanoparticle composites with the addition of different protein disruptors [33]. The freshly prepared milk protein–nisin nanoparticle composite solution was taken, and different protein disruptors were added separately. The concentration of each protein disruptor in the final composite solution was SDS 0.5% (*w*/*v*), urea 6 M, DTT 30 mM, NaCl 0.6 M, SDS 0.5% (*w*/*v*) + urea 6 M, SDS 0.5% (*w*/*v*) + DTT 30 mM, and urea 6 M + DTT 30 mM, and the nanoparticle composite without any added protein disruptors was used as a control. The samples were equilibrated at room temperature for 1 h. The particle size of the solutions was then determined by a Zetasizer Nano ZS instrument, and changes in the visual appearance of the solutions were observed.

### 2.5. Determination of Storage Stability

The storage stability was determined according to Lin et al. [34] with minor modifications. The prepared milk protein–nisin nanoparticle composite was dispensed in glass vials and stored at 25 °C and 4 °C for 28 days, and the particle size, PDI, and changes in appearance were recorded after 0, 7, 14, 21, and 28 days of storage to evaluate its storage stability.

### 2.6. Determination of Emulsification Properties

The emulsification properties were determined according to Sui et al. [35] with slight modifications. First, 30 mL of milk protein solution and milk protein–nisin nanoparticle complex were mixed with 10 mL of soybean oil and homogenized with a high-pressure homogenizer (UltraTurrax T25, IKA Labortechnik, Staufen, Germany) for 2 min at 6250× *g* at room temperature. Then, 100 μL of the emulsion was quickly aspirated from the bottom, added to 5 mL of 0.1% (*w*/*v*) SDS solution, and mixed well, and the absorbance value was measured at 500 nm and recorded as A_0_. After waiting for 10 min, 100 μL of the emulsion was pipetted from the bottom again. The above procedure was repeated, and the absorbance value was recorded as A_10_. The emulsifying activity index (EAI) and emulsion stability index (ESI) were calculated using Equations (1) and (2):

(1) $$EAI\;\left(m^{2}/g\right)=\frac{2\times 2.303\times A_{0}\times D}{N\times C\times 10000}$$

(2) $$ESI\;\left(min\right)=\frac{A_{0}\times 10}{A_{0}-A_{10}}$$

where D represents the dilution factor, which is 50; N represents the volume fraction of oil in the emulsion, which is 0.25 mL/mL; and C represents the initial protein concentration (g/mL) before emulsification.

### 2.7. Determination of Bacteriostatic Activity

#### 2.7.1. Strain Recovery and Activation

Under aseptic conditions, a small amount of glycerol tube-preserved strains was dipped and streaked on nutrient agar plates and incubated overnight at a suitable temperature until single colonies grew. Single colonies were picked, inoculated into 3 mL of nutrient broth medium, and incubated overnight with shaking at an appropriate temperature to obtain an activated bacterial suspension, which was placed in a refrigerator at 4 °C for reserve [36]. A total of 100 μL was taken for bacterial counting by the dilution-coated plate method.

#### 2.7.2. Determination of the Zone of Inhibition

The zone of inhibition was determined using the filter paper disc method [37]. First, 100 μL of bacterial solution (~10^6^ CFU/mL) was evenly spread on a nutrient agar plate and allowed to dry. Then, 6 mm sterile filter papers were placed on the plate coated with the bacterial solution using a tweezer and pressed gently to make it fit tightly on the plate. A 10 μL drop of the sample was aspirated onto the filter paper slice. Sterile PBS was used as a blank control, which was diffused in the refrigerator at 4 °C for 1 h. Then, it was incubated in the incubator at 37 °C for 24 h, after which it was taken out, and the diameter of the zone of inhibition was measured by the criss-cross method.

#### 2.7.3. Determination of the Minimum Inhibitory Concentration (MIC) and Minimum Bactericidal Concentration (MBC)

The MIC and MBC of nisin and milk protein–nisin nanoparticles were determined by the microtiter plate method and double-dilution method [38]. The samples (the initial concentration of nisin was 2.5 mg/mL) were diluted two-fold with nutrient broth medium, and then 100 μL of each was pipetted into a 96-well microplate. Then, 100 μL of bacterial solution (~10^6^ CFU/mL) was mixed with the samples in the microplate. Finally, the microplate was incubated at 37 °C for 24 h. Among them, 100 μL of nutrient broth medium + 100 μL of bacterial solution was used as the positive control, and 100 μL of nutrient broth medium + 100 μL of sterile PBS was used as the negative control. The MIC was defined as the lowest concentration of the samples at which the medium was clarified as seen by the naked eye after incubation at 37 °C for 24 h. Based on the results obtained from the above MIC, 100 μL of samples with concentrations ≥ the MIC were spread on nutrient agar plates and incubated at 37 °C for 24 h. The concentration of sterile growth was determined as the MBC.

#### 2.7.4. Determination of 24 h Short-Term Bacteriostatic Activity

First, 1 mL of bacterial solution (~10^6^ CFU/mL) was mixed with 1 mL of milk protein–nisin nanoparticle composite solution and incubated at 37 °C, 120× *g* on a shaker for 24 h. The samples were taken at 0, 0.5, 1, 2, 4, 6, 9, 12, 16, 20, and 24 h, and then colony coating counting was performed [39]. The same concentration of nisin solution, as well as sterile PBS, were set as control groups for the same operation as above to observe the 24 h short-term bacteriostatic activity of milk protein–nisin nanoparticles.

#### 2.7.5. Determination of 7 d Long-Term Bacteriostatic Activity

First, 5 mL of bacterial solution (~10^6^ CFU/mL) was mixed with 5 mL of milk protein–nisin nanoparticle composite solution and incubated at 37 °C, 120× *g* on a shaker. After that, 100 μL of the solution was taken every 1 d for gradient dilution to the appropriate concentration, and then the colonies were coated and counted for 7 d. The control groups were set up, and the same concentration of nisin solution and sterile PBS were used for the same operation as above to observe the 7 d long-term bacteriostatic activity of the milk protein–nisin nanoparticles.

### 2.8. Effect of Milk Protein–Nisin Nanoparticles on Bacterial Cell Membranes

#### 2.8.1. Determination of Leakage of Bacterial Contents

The effect of milk protein–nisin nanoparticles on the cell membrane integrity of *L. monocytogenes*, *S. aureus*, and *B. cereus* was investigated by determining the content of bacterial extracellular nucleic acids and proteins [39]. The bacterial solution in the logarithmic growth period was centrifuged at 5000× *g* for 5 min, washed three times with sterile PBS, and resuspended to a concentration of ~10^8^–10^9^ CFU/mL. Then, 1 mL of the resuspended bacterial solution was mixed with 1 mL of milk protein–nisin nanoparticle composite solution and incubated on a shaking bed at 37 °C, 120× *g*. The samples were taken at 0, 1, 2, 4, 6, 8, and 10 h and centrifuged (5000× *g*, 5 min), and the supernatant was filtered through a 0.22 μm filter and stored at 4 °C for use. In the control group, the same concentration of nisin solution and sterile PBS were used in the same operation, and the nucleic acid and protein contents in the supernatant were determined by an ultra-micro spectrophotometer (NanoDrop 2000, Thermo Fisher Scientific, Waltham, MA, USA).

#### 2.8.2. Observation of Bacterial Micromorphology

SEM was used to observe the micromorphologic changes in *L. monocytogenes*, *S. aureus*, and *B. cereus* before and after L-N treatment [39]. Bacteria cultured to the logarithmic stage were taken, added to the L-N solution (*v*:*v* = 1:1), and incubated at 37 °C, 120× *g* for 1 h. The addition of sterile PBS was used as a blank control. Then, the bacterial solution was centrifuged (5000× *g*, 5 min) to obtain the bacterial precipitate and then washed with sterile PBS three times, and 2.5% glutaraldehyde was added to fix it at 4 °C for 4 h. The fixed bacteria were washed again with sterile PBS three times and then dehydrated by gradient with different concentrations of ethanol (30%, 50%, 70%, 85%, 95%, and 100% × 2) for 15 min. Finally, the bacterial precipitate was resuspended with 200 μL of anhydrous ethanol and then vacuum freeze-dried. The dried bacteria were evenly coated onto the conductive adhesive, sprayed with gold, and then observed.

### 2.9. Statistical Analysis

All tests were repeated three times, and the results were reported as mean ± standard deviation. The experimental data were statistically analyzed using Origin 2021 and IBM SPSS Statistics 22.0 software. Between-group comparisons were performed using one-way analysis of variance (ANOVA), with the post hoc Duncan test for multiple comparisons. *p* < 0.05 was considered to indicate statistical significance.

## 3. Results and Discussion

### 3.1. Particle Size and Apparent Morphology Observation

**Figure 1:**
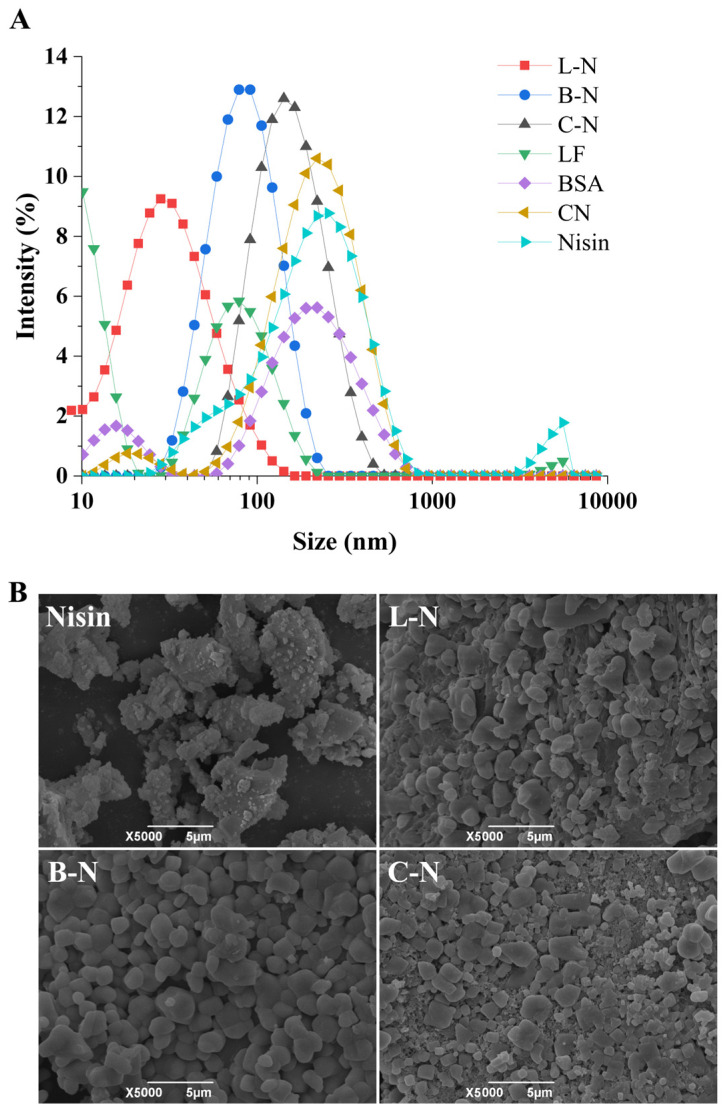
Morphological structure of different samples. (**A**) Particle size distribution. (**B**) SEM characterization. L-N: lactoferrin–nisin nanoparticles, B-N: bovine serum albumin–nisin nanoparticles, C-N: casein–nisin nanoparticles.

Particle size is an important parameter of nanoparticles. The size of a particle can affect many of its properties, such as physicochemical properties, storage stability, and bioavailability. Usually, nanoparticles with a small particle size have more unique functional properties than those with a larger particle size [40]. In this study, we used particle size distribution (Figure 1A) and SEM (Figure 1B) to characterize the morphological structure of different samples.

As can be seen from Figure 1A, the particle sizes of the three nanoparticles (L-N, B-N, C-N) were smaller than that of nisin, and all of them showed a single-peak distribution with a narrower peak shape, which suggested that the nanoparticles were more compact in structure and more stable. This difference also meant that the measured particle size distribution came from the nanoparticle complex rather than a single component, further demonstrating that effective complexation of milk protein–nisin nanoparticles occurred [41]. Among them, L-N had the smallest particle size of about 29.83 nm, which was significantly smaller than the high-methoxy pectin oligosaccharide (HMPOS)-A/nisin nanoparticles (308.0 nm) and HMPOS-B/nisin nanoparticles (319.2 nm) prepared by Wang et al. [41].

Figure 1B shows that nisin was irregularly shaped and had a rough surface with voids of different sizes [41]. The SEM image of L-N shows that LF and nisin were in a state of orderly cross-linking, forming a dense mesh structure with a full and rounded surface and fewer voids [42]. The surface of B-N was relatively flat, which was presumed to be due to the fact that the BSA, as a kind of globular protein, was encapsulated on the surface of nisin. The SEM image of C-N shows that the incorporation of CN filled the voids of nisin to a certain extent, but it still showed an apparent irregular shape, with more voids on the surface, uneven distribution, and larger particles.

### 3.2. Analysis of Molecular Interactions among Nanoparticles

**Figure 2:**
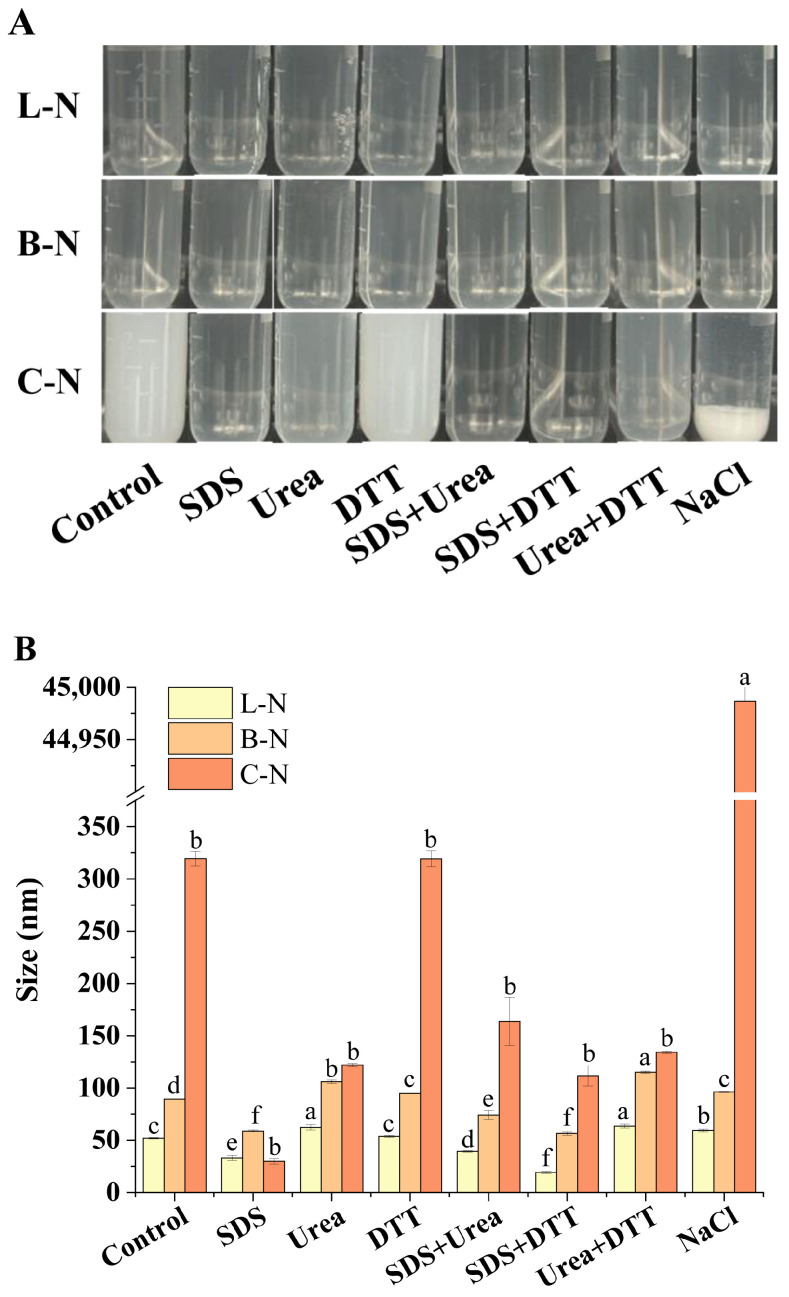
Effect of various protein disruptors on different samples. (**A**) Visual appearance. (**B**) Average particle size. L-N: lactoferrin–nisin nanoparticles, B-N: bovine serum albumin–nisin nanoparticles, C-N: casein–nisin nanoparticles. Values with different superscript lowercase letters in the same row are significantly different (*p* < 0.05).

In order to explore the role of different forces in the formation and maintenance of milk protein–nisin nanoparticles, we analyzed the changes in particle size and visual appearance of nanoparticle complexes after adding different protein disruptors (Figure 2).

Among the protein disruptors used, SDS mainly disrupted hydrophobic interactions, urea had excellent effects in disrupting hydrogen bonds, DTT could reduce disulfide bonds in proteins, and NaCl could shield electrostatic interactions [43].

As shown in Figure 2A, the various protein disruptors had no obvious effect on the visual appearance of L-N. Further comparison of the changes in particle size (Figure 2B) revealed that the presence of SDS alone or in combination resulted in a significant decrease in L-N particle size, suggesting that the disruption of hydrophobic interactions could dissociate L-N into smaller particles with stronger hydrophobic interactions. The presence of urea, on the other hand, resulted in a significant increase in the particle size of L-N, which could be attributed to the breaking of hydrogen bonds, stretching out the external structure of the nanoparticles. However, the presence of DTT and NaCl had less effect on the L-N particle size compared with SDS and urea, suggesting that disulfide bonding and electrostatic interactions played a minor role. The effect of each protein disruptor on B-N was observed, and the results were found to be similar to those of L-N. The above results indicated that in L-N and B-N, hydrophobic interactions were the main force that maintained their internal structure, and hydrogen bonding was the main force that maintained their external structure [33,43]. It can be seen in Figure 2 that upon the addition of NaCl, C-N exhibited significant aggregation and an increase in particle size, indicating that electrostatic interactions play a key role in the formation of C-N structures and the stability of particles. It is well known that casein tends to aggregate in NaCl, suggesting that this force may primarily be provided by casein within C-N. DTT alone did not cause obvious changes in C-N, and the use of SDS and urea alone or in combination decreased the turbidity of C-N. This indicated that hydrophobic interactions and hydrogen bonding also contribute to the stability of C-N [44].

### 3.3. Spectral Analysis

#### 3.3.1. Functional Group Analysis

**Figure 3:**
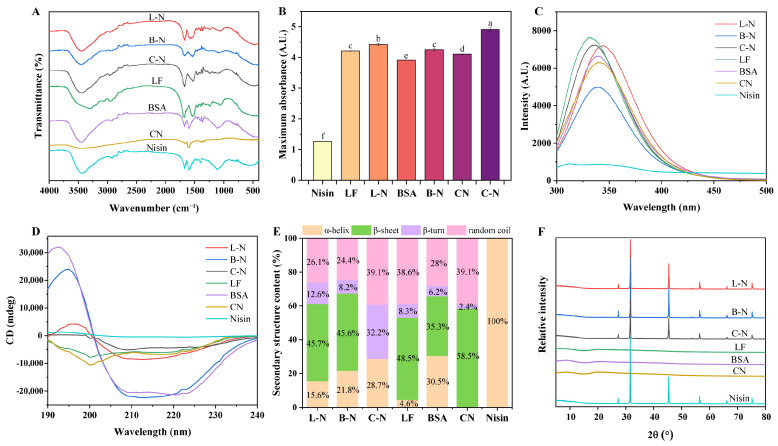
Spectral maps of different samples. (**A**) FTIR spectra; (**B**) maximum absorption intensity at 225 nm; (**C**) fluorescence spectra; (**D**) CD spectra; (**E**) secondary structure content; and (**F**) XRD spectra. L-N: lactoferrin–nisin nanoparticles, B-N: bovine serum albumin–nisin nanoparticles, C-N: caseins–nisin nanoparticles. Values with different superscript lowercase letters in the same row are significantly different (*p* < 0.05).

FTIR was a valuable method for analyzing specific functional groups and structural changes in the substances (Figure 3A).

The FTIR spectrum of nisin showed three characteristic absorption peaks at 3440 cm^−1^, 1670 cm^−1^, and 1590 cm^−1^, similar to the study of Krivorotova et al. [45]. Among these, 3440 cm^−1^ was attributed to the O-H stretching vibration of -COOH, 1670 cm^−1^ to the C=O stretching vibration of the amide I band, and 1590 cm^−1^ to the N-H bending vibration and C-N stretching vibration of the amide II band [46,47]. It was observed that the characteristic peaks of nisin at 3440 cm^−1^ were all shifted to higher wave numbers (3450 cm^−1^) after nisin was complexed with milk proteins, which might be speculated to be due to the formation of new intermolecular hydrogen bonds between the two [48]. Both C=O and C-N stretching vibrations were associated with hydrogen bonding between the secondary structure units of proteins, and if they were shifted, it meant that they were interacting with each other [49]. As can be seen in Figure 3A, among the three nanoparticles, the characteristic peaks of nisin at 1670 cm^−1^ were not shifted, but the characteristic peaks at 1590 cm^−1^ were all shifted to lower wavelengths, L-N to 1580 cm^−1^, B-N to 1540 cm^−1^, and C-N to 1530 cm^−1^, which also suggested that nisin interacted with the milk proteins to promote the formation of hydrogen bonds [50].

#### 3.3.2. UV Characteristic Analysis

Analyzing the UV–vis absorption spectra made it possible to identify the structural changes of nisin after its complexation with milk proteins. Figure 3B shows the UV maximum absorption intensity of milk proteins, nisin, and its nanoparticles at 225 nm. The UV–vis spectrum of nisin showed a major peak located in the far UV region (225 nm), reflecting the peptide backbone structure, similar to that reported by Abid et al. [48]. In general, the transformation of the π-π* peptide backbone structure could be characterized by UV–vis absorption peaks in the low wavelength region [51]. However, nisin had no characteristic absorption peak at 280 nm because it did not contain aromatic amino acids [52]. As shown in Figure 3B, the maximum absorption intensities of all three nanoparticles at 225 nm were significantly increased compared with those of nisin and milk proteins, which might be attributed to the formation of new intermolecular hydrogen bonds between milk proteins and nisin and induced changes in the peptide conformation of nisin, such as α-helix, β-sheet, β-turn, and random coil contents [48].

#### 3.3.3. Fluorescence Characteristic Analysis

Since proteins contain amino acids such as tryptophan (Trp), tyrosine (Tyr), and phenylalanine (Phe), which can be excited at a certain wavelength to produce endogenous fluorescence, when the structure of a protein or polypeptide is altered, such as folded, stretched, or combined with other substances, its fluorescence intensity or maximum emission wavelength will change [53]. Therefore, in this study, we analyzed whether the fluorescent groups in the proteins were altered by the change in fluorescence spectra so as to further analyze the structural changes in the milk proteins after complexing with nisin (Figure 3C). As can be seen in Figure 3C, nisin showed a small amount of fluorescence signal in the range of 300–500 nm, which did not interfere with the protein fluorescence signal. The maximum emission wavelength of LF was around 331 nm, while the maximum emission wavelength of L-N was around 345 nm, showing a red shift. This phenomenon suggested that the complexation of LF and nisin changed the structure of LF to some extent, resulting in more side chains of LF being exposed to the environment and the Trp surroundings being more hydrophilic with increased polarity [54]. Meanwhile, the fluorescence intensity of L-N decreased because the fluorescent groups were partially encapsulated when LF was complexed with nisin, which led to a decrease in fluorescence intensity. The fluorescence intensity of BSA decreased after complexing with nisin, but the maximum emission wavelengths did not shift, and they were all around 339 nm. This result indicated that there was an interaction between BSA and nisin, but this interaction did not change the polar environment of BSA. It can also be seen in Figure 3C that the maximum emission wavelength of CN was around 339 nm, while the maximum emission wavelength of C-N was slightly blue-shifted, around 336 nm, and the fluorescence intensity of C-N was enhanced. Usually, folded proteins had lower fluorescence wavelengths and higher fluorescence intensities, which might be related to the solvent environment of Trp. When the solvent polarity around the Trp decreased, the maximum emission wavelength decreased to lower wavelengths, resulting in an increase in fluorescence intensity [55].

#### 3.3.4. Secondary Structural Analysis

CD spectroscopy is often used to detect changes in the secondary structure content of proteins [56]. Therefore, CD spectroscopy was used in this study to determine and analyze the changes in the secondary structure of milk proteins and nisin before and after recombination (Figure 3D,E, Table 1).

As can be seen in Table 1, the complexation with nisin significantly increased the content of α-helix and β-turn of LF from 4.6% to 15.6% and 8.3% to 12.6%, respectively, whereas the content of random coil was reduced, which was associated with an enhancement in the positive Cotton effect in the CD spectra (Figure 3D), proving that the addition of nisin caused the structure of LF to become relatively stable [57]. The CD spectra of BSA and B-N showed one positive characteristic peak at 190–200 nm and two negative characteristic peaks at 200–230 nm, which corresponded to the characteristic peaks of α-helix and β-sheet, suggesting that the secondary structures of both were dominated by α-helix and β-sheet [58]. However, the complexation of BSA with nisin resulted in a significant decrease in α-helix content from 30.5% to 21.8% and an increase in β-helix and β-turn content, suggesting that the complexation of BSA with nisin induced the unfolding of the BSA structure, leading to a decrease in stability [58]. In the secondary structure of CN, the content of α-helix was 0%, but after complexation with nisin, the content of α-helix was significantly increased to 28.7%, and it was hypothesized that the increased α-helix came from nisin.

#### 3.3.5. Crystallinity Analysis

The crystalline nature of milk proteins, nisin, and its nanoparticles were analyzed by XRD (Figure 3F). It was seen that nisin had seven characteristic diffraction peaks at 2θ = 27.29, 31.64, 45.37, 53.78, 56.40, 66.15, and 75.22°, which is in accordance with the study of Lin et al. [34]. This was mainly due to its compact molecular structure as well as neat crystalline structure, whereas none of the three proteins had characteristic diffraction peaks, indicating that milk proteins had an amorphous structure. As can be seen in Figure 3F, when milk proteins were complexed with nisin, its nanoparticles showed the same characteristic diffraction peaks as those of nisin, indicating that the formation of nanoparticles did not destroy the crystal structure of nisin [34]. However, their peak intensities were all weakened to different degrees, probably because of the existence of hydrogen bonding and hydrophobic interactions between nisin and milk proteins, which reduced the crystallinity of nisin and thus led to the weakening of the intensity of the diffraction peaks, which proved the successful formation of the milk protein–nisin nanoparticle complexes [59].

### 3.4. Stability Analysis

#### 3.4.1. Thermal Stability

**Figure 4:**
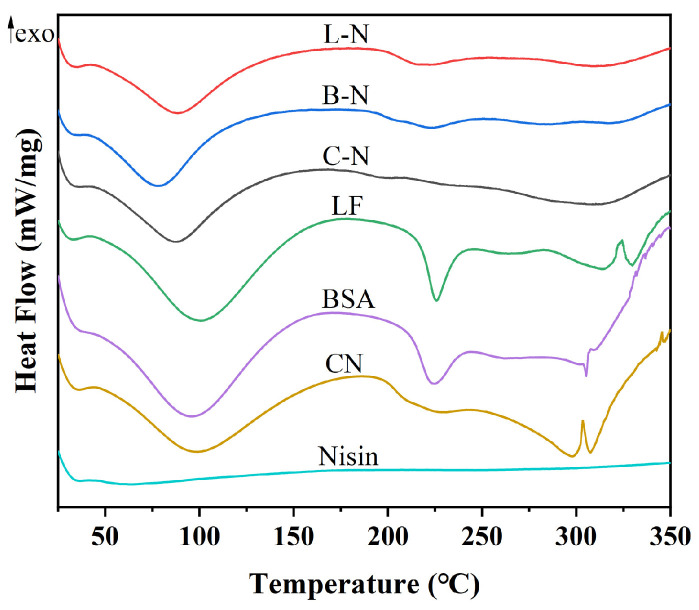
DSC spectra of different samples. L-N: lactoferrin–nisin nanoparticles, B-N: bovine serum albumin–nisin nanoparticles, C-N: casein–nisin nanoparticles. The direction of the arrow indicates exothermic.

DSC was used to analyze the thermodynamic properties of milk protein–nisin nanoparticles, and the results are shown in Figure 4.

As shown in Figure 4, nisin did not show any obvious heat absorption or exothermic peaks, indicating that nisin was thermally stable. Four heat absorption peaks appeared at 100, 226, 315, and 330 °C for LF, four heat absorption peaks appeared at 95, 224, 305, and 310 °C for BSA, and four heat absorption peaks appeared at 98, 220, 298, and 307 °C for CN, of which the peaks around 100 °C might be caused by the evaporation of water in the samples [60]. However, in the DSC plots of the milk protein–nisin nanoparticles, only one heat-absorption peak at around 100 °C appeared. The other characteristic peaks of milk protein did not appear, presumably because of the interaction between milk protein and nisin, which led to the loss of the structure of the milk protein, and possibly because of the improved thermal stability of the nanoparticle complexes, which did not show the heat-absorption peaks at the high temperatures [61].

#### 3.4.2. Storage Stability

**Figure 5:**
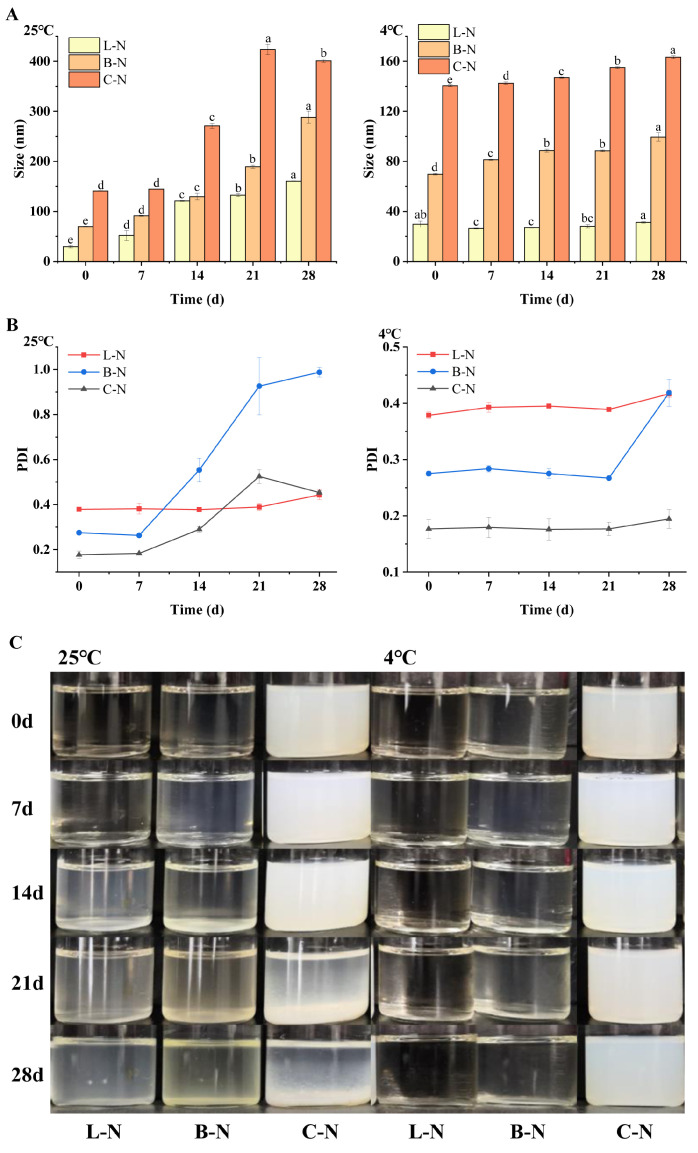
Changes in different samples during storage at 25 °C and 4 °C. (**A**) Particle size; (**B**) PDI; and (**C**) visual appearance. L-N: lactoferrin–nisin nanoparticles, B-N: bovine serum albumin–nisin nanoparticles, C-N: casein–nisin nanoparticles. Values with different superscript lowercase letters in the same row are significantly different (*p* < 0.05).

Storage stability is an important indicator of the stability and reliability of a developed material during storage [62]. Generally, particle size and PDI are the key indicators to characterize the stability of nanoparticles [63], so the freshly prepared milk protein–nisin nanoparticles were stored at 25 °C and 4 °C for 28 days. The changes in particle size and PDI were monitored during the storage process, as well as the changes in visual appearance were recorded, and the results are shown in Figure 5.

As can be seen in Figure 5A, the particle size of nanoparticles showed a gradual increase with the extension of storage time at 25 °C, and the particle size increased slowly in the first 7 d and rapidly after that. In addition, the PDI of nanoparticles (Figure 5B) also showed a similar trend as the particle size under the same storage condition [64]. This suggested that the nanoparticles could maintain a relatively stable structure for 7 d at 25 °C. Among them, the particle size and PDI of L-N changed more gently than that of B-N and C-N, indicating that L-N had the best storage stability. Observing the changes in particle size and PDI of nanoparticles at 4 °C, it was found that they also showed a slow growth trend. However, compared with 25 °C, under the same placement time, the changes in the parameters of nanoparticles at 4 °C were much smaller than those at 25 °C, and the trend was gentler [62], among which the particle size of L-N was almost unchanged, which proved that the stability of L-N storage was the best once again. The visual appearance changes in the nanoparticles during storage (Figure 5C) were found to be consistent with the results of particle size and PDI. Compared with the samples at day 0, it was observed that L-N and B-N gradually became turbid with increasing storage time at 25 °C, with L-N changing more slowly, and no significant change was observed during 7 d. The color of B-N gradually changed to yellow from day 7 and eventually formed a precipitate, while C-N was observed as a significant precipitate at the bottom after 21 d of storage. However, no obvious changes were observed in all three nanoparticle complexes under 4 °C storage conditions. The same trend was observed in the complexation of nisin with fucoidan, i.e., the zeta potential and hydrodynamic diameter of the nanoparticles did not change significantly in most cases after 4 weeks of storage at 4 °C [65].

In summary, the results showed that the storage environment at 4 °C was more suitable for the milk protein–nisin nanoparticles. Therefore, in order to maintain the stability of the nanoparticles, it was necessary to store them at 4 °C conditions. In addition, among the three nanoparticles, L-N had the best storage stability.

#### 3.4.3. Emulsifying Properties

**Figure 6:**
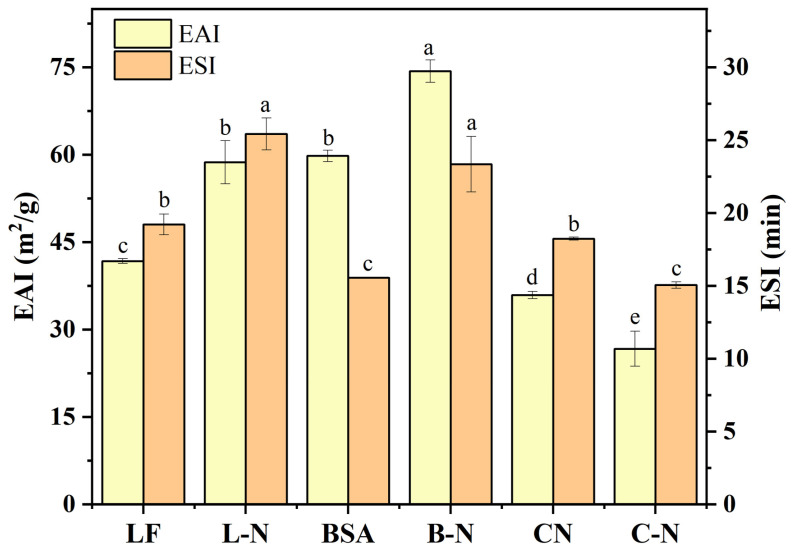
EAI and ESI of different samples. L-N: lactoferrin–nisin nanoparticles, B-N: bovine serum albumin–nisin nanoparticles, C-N: casein–nisin nanoparticles. Values with different superscript lowercase letters in the same row are significantly different (*p* < 0.05).

Protein emulsification activity refers to the oil–water interfacial area per unit mass of protein that could be stabilized, and emulsification stability refers to the emulsification property of proteins that maintained oil–water mixing and did not separate within a predetermined time, reflecting the strain resistance to external conditions [66]. The emulsifying activity and emulsion stability of proteins are of great importance in food applications. Figure 6 shows the changes in emulsification activity and emulsion stability before and after the combination of milk proteins and nisin.

As can be seen in Figure 6, the emulsification activity and emulsification stability of LF and BSA significantly increased after compounding with nisin, among which the emulsification activity of L-N increased most significantly by 40.56% compared with LF, while the emulsification stability increased 32.29%. This was firstly due to the structure of nisin, which had hydrophobic residues and lipid–water amphiphilic properties; thus, nisin had the ability to improve the emulsification properties of solutions [67]. Moreover, nisin was positively charged, and these charged residues could form hydrogen bonds with water molecules as well as intermolecular interactions with other proteins in solution. It is well known that LF is positively charged [68], and it self-assembled with nisin to form L-N, which led to an increase in the positive surface charge and further strengthened the electrostatic repulsion among the particles in solution, which could enhance the emulsion stability. Proteins had better emulsification properties at a pH away from the pI [69]. Therefore, the improvement in the emulsification performance of BSA might be mainly provided by nisin. However, the emulsifying activity and emulsification stability of CN decreased after compounding with nisin. CNs were the proteins with negative surface charge. CNs and nisin self-assembled to form C-N, and the surface charge of both were neutralized. Research has shown that the more negatively or positively charged, the more the net charge increases and the stronger the repulsive effect, which helps to produce better emulsification performance [70]. However, the electrostatic repulsive effect on C-N decreased, showing a tendency to agglomerate and sink and, therefore, a decrease in the emulsification properties.

### 3.5. Bacteriostatic Activity of Nanoparticles

#### 3.5.1. Bacteriostatic Circle

**Figure 7:**
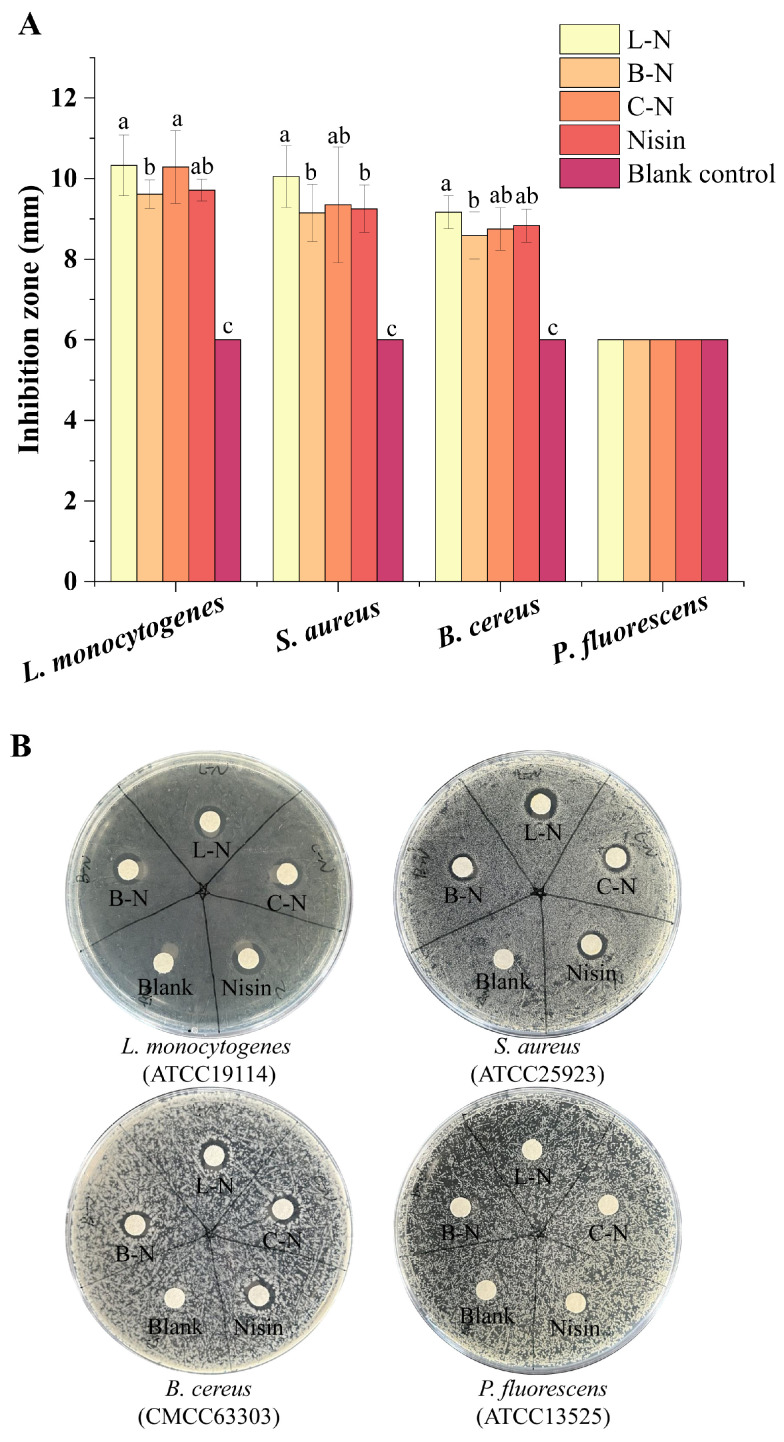
The inhibition zone of different samples against four strains. (**A**) Antibacterial zone diameter. (**B**) Visual appearance. L-N: lactoferrin–nisin nanoparticles, B-N: bovine serum albumin–nisin nanoparticles, C-N: casein–nisin nanoparticles. Values with different superscript lowercase letters in the same row are significantly different (*p* < 0.05).

Since the previous experiments confirmed that the three proteins (LF, BSA, CN) have no bacteriostatic effect, the subsequent bacteriostatic experiments were conducted only on nisin and its nanoparticles. The specific experimental data are shown in Figure S1 of the Supporting Information. The antibacterial activity of the milk protein–nisin nanoparticles against four different bacteria was observed by measuring the diameter of the antibacterial zone, as shown in Figure 7.

The three nanoparticles and nisin showed significant bacteriostatic effects against the three Gram-positive bacteria (*L. monocytogenes*, *S. aureus*, *B. cereus*) but not against the Gram-negative bacteria (*P. fluorescens*). As a cationic antimicrobial peptide, nisin has been reported to show relatively broad antibacterial activity against Gram-positive bacteria. It acts mainly through electrostatic interaction with negatively charged phospholipids, increasing membrane permeability through pore formation, leading to the rapid efflux of essential small molecules from the cells, and interfering with the biosynthesis of the cell wall, which in turn leads to the death of the bacteria. The large amount of lipopolysaccharide attached to the cell wall surface of Gram-negative bacteria could restrict nisin, which contains numerous hydrophobic amino acids, from penetrating through the cell wall of Gram-negative bacteria, which made the inhibitory effect of nisin on Gram-negative bacteria poorer [71].

#### 3.5.2. MIC and MBC

The zone of inhibition experiment showed that the milk protein–nisin nanoparticles and nisin had no inhibitory effect on Gram-negative bacteria, so the subsequent experiments were only carried out for further in-depth studies on the above three Gram-positive bacteria. Table 2 shows the MIC and MBC of the milk protein–nisin nanoparticles and nisin against the three Gram-positive bacteria.

It was found that the MIC and MBC of L-N and B-N against the three Gram-positive bacteria were the same, indicating that they had similar inhibitory activities. The MIC and MBC of C-N against the three Gram-positive bacteria were higher than those of L-N and B-N. The MBC of C-N against *B. cereus* was not observed in the range of the set concentration, indicating that its inhibitory activity was the worst. According to the SEM of C-N, most of the nisin adhered to the surface of the particles, resulting in its initial rapid release of nisin, which could be sustained. Later, because of the inactivation of nisin and bacterial resistance, the bacteria gradually grew [39]. Therefore, the concentration of C-N needed to be increased to achieve the same inhibitory effect.

#### 3.5.3. Twenty-Four-Hour Short-Term Bacteriostatic Activity and 7 d Long-Term Bacteriostatic Activity

**Figure 8:**
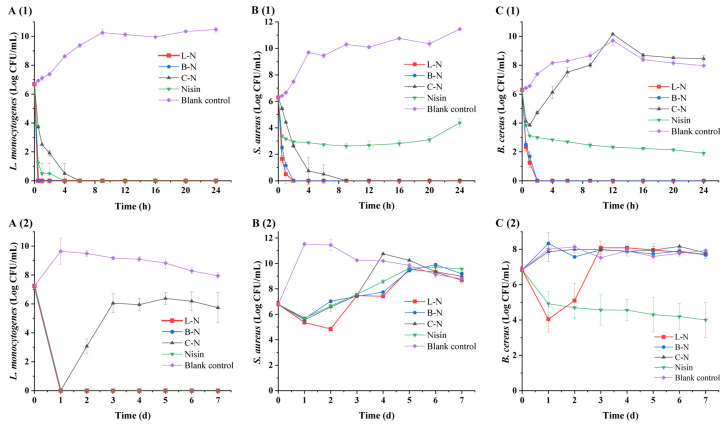
Bacteriostatic activity of different samples. (**A**) *L. monocytogenes*; (**B**) *S. aureus*; and (**C**) *B. cereus* (**1**) 24 h short-term bacteriostatic activity and (**2**) 7 d long-term bacteriostatic activity. L-N: lactoferrin–nisin nanoparticles, B-N: bovine serum albumin–nisin nanoparticles, C-N: casein–nisin nanoparticles.

When an inhibitory substance was present, the short-term bacteriostatic activity and long-term bacteriostatic activity of the milk protein–nisin nanoparticles and nisin were analyzed and evaluated by determining the growth of three Gram-positive bacteria over a period of 24 h and 7 d, respectively (Figure 8).

As can be seen in Figure 8A(1), L-N and B-N effectively inhibited the growth of *L. monocytogenes* at 0.5 h (colony number of 0 CFU/mL), while nisin and C-N achieved this effect only at 4 h and 6 h, respectively. After that, no growth of *L. monocytogenes* was observed up to 24 h, which indicated that nanoparticles and nisin had fast sterilization and a good inhibition effect on *L. monocytogenes*. For *S. aureus* (Figure 8B(1)), L-N and B-N effectively inhibited its growth to 0 CFU/mL at 2 h, and L-N sterilized it faster, while C-N took 9 h, after which no growth of *S. aureus* was observed. Nisin rapidly reduced the colony number of *S. aureus* to about 10^2^ CFU/mL at 2 h. The colony number remained stable thereafter until *S. aureus* began to grow slowly at 12 h, and a colony size of 10^4^ CFU/mL was detected at 24 h. As observed in Figure 8C(1), C-N had a rapid sterilization effect on *B. cereus* in the first 1 h, after which the number of colonies increased rapidly and even surpassed the blank group at 12 h. On the other hand, nisin was rapidly sterilized in the first 1 h and then slowly sterilized until the number of colonies was detected to be around 10^2^ CFU/mL at 24 h. In the case of L-N and B-N, the growth of the colonies could not be detected at 2 h, and the sterilization rate of L-N was faster than B-N.

As can be seen in Figure 8A(2), L-N, B-N, C-N, and nisin inactivated *L. monocytogenes* to undetectable levels on day 1, and it was observed that the samples were still effective against *L. monocytogenes* on day 7, except for C-N. For *S. aureus* (Figure 8B(2)), L-N was able to effectively inhibit the growth of *S. aureus* for 2 days, with a reduction in bacterial counts of about 10^2^ CFU/mL, after which the activity was lost and *S. aureus* began to grow was indistinguishable from the blank control on day 5. B-N, C-N, and nisin were able to effectively inhibit the growth of *S. aureus* on day 1, with a reduction in bacterial counts by about 10^1^ CFU/mL, after which *S. aureus* grew gradually, with the C-N group growing to be indistinguishable from the blank control on day 4 and the B-N and nisin groups growing to be indistinguishable from the blank control on day 5. For *B. cereus* (Figure 8C(2)), it was found that only L-N among the nanoparticles had an inhibitory activity against it. L-N led to a significant decrease in bacterial concentration within 1 d and continued to have inhibitory activity for 2 days, significantly slowing down the growth of *B. cereus*.

In summary, the results showed that the short-term bacteriostatic activity and long-term bacteriostatic activity of the three nanoparticles were good, and the magnitude of the bacteriostatic activity of the three nanoparticles was L-N > B-N > C-N. Among them, the antimicrobial effect of L-N was prominent, which could effectively inhibit the growth of *L. monocytogenes*, *S. aureus*, and *B. cereus*. Nanoparticles are considered a protective barrier for nisin and can control its release, which has been proven to enable nisin to produce more effective antibacterial effects [72]. The release of nisin from nanoparticles can be divided into two stages as follows: the initial rapid-release stage and the subsequent slow-release stage [59]. The rapid release in the initial stage was due to the large amount of nisin released from the surface of nanoparticles, resulting in the rapid antibacterial effect in this stage. Subsequently, because of the ordered cross-linked network structure of L-N, the structure was relatively tight and could sustainably and stably release nisin from the interior of nanoparticles. The sustained release effect was better, so its antibacterial effect was more effective [73].

### 3.6. Effect of Milk Protein–Nisin Nanoparticles on Bacterial Cell Membranes

#### 3.6.1. Analysis of Bacterial Content Leakage

**Figure 9:**
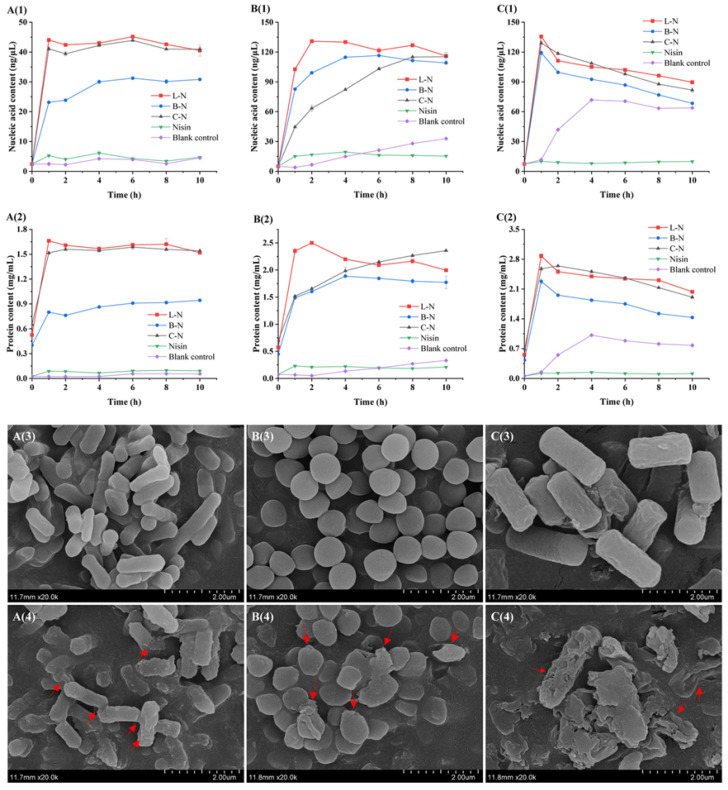
Effect of different samples on bacterial cell membranes. (**A**) *L. monocytogenes*; and (**B**) *S. aureus*; (**C**) *B. cereus* showing the extracellular nucleic acid content (**1**) and protein content (**2**) of the three Gram-positive bacteria after different sample treatments. SEM images of the three Gram-positive bacteria before (**3**) and after (**4**) L-N treatment. L-N: lactoferrin–nisin nanoparticles, B-N: bovine serum albumin–nisin nanoparticles, C-N: casein–nisin nanoparticles.

The cell membrane, as a key structural component of bacteria, provides a stable internal environment suitable for the survival of bacteria, which can prevent the outflow of nucleic acids, proteins, and other macromolecules inside the cell and maintain the life activities of bacteria, such as growth, reproduction, genetics, and metabolism [74]. Therefore, the leakage of intracellular substances can be used as a typical indicator to judge the integrity of the cell membrane [75]. The integrity of the bacterial cell membrane was investigated by measuring the leakage of nucleic acids and proteins in the cells of the three Gram-positive bacteria after different sample treatments, and the results are shown in Figure 9A(1)–C(1),A(2)–C(2).

As can be seen in Figure 9A(1)–C(1),A(2)–C(2), the nucleic acid and protein content in the control group was very low at 0 h and increased thereafter, but without a significant trend. In contrast, the nucleic acid and protein content in the *B. cereus* control group increased slowly after 1 h. This might be due to the changes in the survival environment caused by nutrient deficiencies, which subsequently caused bacterial cell autolysis and death. The content of nucleic acids and proteins in the experimental group was initially low, and with the prolongation of the nanoparticles’ action time, the content of nucleic acids and proteins was significantly higher compared with the control group. Among them, the highest nucleic acid and protein contents were leaked by the three Gram-positive bacteria under the effect of L-N (the nucleic acid and protein leakage was 45.17 ng/μL and 1.66 mg/mL for *L. monocytogenes*, 130.92 ng/μL and 2.50 mg/mL for *S. aureus*, and 135.65 ng/μL and 2.88 mg/mL for *B. cereus*, respectively), and the shortest action time was required to reach the maximum. In summary, the leakage of intracellular components in the experimental group was much higher than that in the control group, indicating that the milk protein–nisin nanoparticles made the cell membranes of the three Gram-positive bacteria increase in permeability, leading to the efflux of the cellular contents, and ultimately causing the death of the bacteria, where L-N had the most pronounced effect and the fastest rate of action. This might be due to the fact that L-N had the smallest particle size, which gave it a larger surface area and high affinity for bacteria cells, resulting in a quantum size effect that enhanced the bacterial inhibition of L-N [76].

#### 3.6.2. Analysis of Bacterial Micromorphology

Compared with the determination of the leakage of nucleic acids and proteins in the bacterial cell, SEM can more intuitively reflect the changes in the morphology of bacterial cells as a means of observing the integrity of the bacterial cell membrane. In this study, L-N was used as a representative of the milk protein–nisin nanoparticles. It was applied to *L. monocytogenes*, *S. aureus*, and *B. cereus*, and SEM was utilized to observe the changes in the morphology of the bacteria before and after they were treated with L-N. The results are shown in Figure 9A(3)–C(3),A(4)–C(4)

Figure 9A(3) shows an SEM image of *L. monocytogenes* before L-N treatment, which indicates that the cells of the bacterium were closely arranged, the structure of the bacterium was natural and complete, and the surface was flat and smooth. Figure 9A(4) shows an SEM image of *L. monocytogenes* after L-N treatment, which indicates that the surface of *L. monocytogenes* became rough and uneven, with an obvious wrinkling phenomenon and adhesion between cells. Figure 9B(3) shows an SEM image of *S. aureus* before L-N treatment, which indicates that the bacterium was obviously spherical, its cells were full and round, the surface was smooth, and the boundaries between cells were clear. Figure 9B(4) shows an SEM image of *S. aureus* after L-N treatment, which indicates that at this time, the boundaries between the cells became blurred, the bacterial body became flattened and showed an irregular shape, some of the bacterial body was obviously ruptured, there were accumulations on the surface of the bacterial body, and it even adhered to form a clump. Figure 9C(3) shows an SEM image of *B. cereus* before L-N treatment, which indicates that the bacterial cells were overall fuller, with clear contours and short rods, and no obvious abnormality. Compared with the control group, L-N-treated *B. cereus* (Figure 9C(4)) showed significant differences in bacterial morphology. The outline of the bacterium became blurred, the cells underwent obvious wrinkling or were even broken and lysed, the cell contents flowed out, and the cell morphology was severely damaged. The cell morphology of the bacteria was damaged because the inhibitory mechanism of nisin mainly acted on the cell membrane of the bacteria, causing the formation of pores and, ultimately, the death of the bacteria [14].

The above results indicated that L-N had good antibacterial effects on *L. monocytogenes*, *S. aureus*, and *B. cereus*. It could damage the integrity of the bacterial cell membranes or even cause them to be completely lost, resulting in irreversible damage to the bacteria. In addition, it showed important development potential in the prevention and control of bacterial contamination.

## 4. Conclusions

In this study, we successfully synthesized different milk protein–nisin nanoparticles loaded with nisin by utilizing the self-assembling properties of three milk proteins, namely, LF, BSA, and CN. By studying a series of properties of milk protein–nisin nanoparticles, we found that the biological activity of nisin was still retained after complexing with the milk proteins, and L-N achieved the best performance among the three kinds of nanoparticles, namely, L-N, B-N, and C-N. L-N was a relatively dense reticular structure with the smallest particle size (29.83 ± 2.42 nm) and good stability. By measuring the interaction forces, it was found that the hydrophobic interaction was the main force that maintained the internal structure of L-N, and hydrogen bonding was the main force that maintained its external structure. The results of the bacteriostatic test showed that L-N had outstanding bacteriostatic performance against *L. monocytogenes*, *S. aureus*, and *B. cereus*. Its antibacterial process was mainly divided into two phases, the initial rapid antibacterial phase, followed by the continuous slow antibacterial phase, which was mainly due to the fact that the release of nisin from the nanoparticles could be divided into two phases as follows: the initial rapid-release phase and the subsequent slow-release phase. The rapid release in the initial phase was due to the release of a large amount of nisin from the surface of the nanoparticles, resulting in a rapid antimicrobial effect in this phase. Subsequently, the continuous and stable release of nisin inside L-N resulted in a better slow release, and thus its antimicrobial effect was more effective. L-N mainly acted on the cell membrane of bacteria, causing its permeability to change and resulting in the outflow of bacterial contents, which ultimately led to the death of bacteria. The above results indicated that L-N had good properties and significant bacteriostatic activity against foodborne pathogens. Therefore, this study lays the foundation for the practical application of L-N and suggests its potential for use as a new type of food preservative, which can help to improve the bioavailability of nisin in the food system.

## Figures and Tables

**Table 1:** Secondary structure content of different samples.

Samples	α-Helix (%)	β-Sheet (%)	β-Turn (%)	Random Coil (%)
L-N	15.6 ± 1.1 ^d^	45.7 ± 4.5 ^b^	12.6 ± 2.3 ^b^	26.1 ± 1.0 ^bc^
B-N	21.8 ± 0.1 ^c^	45.6 ± 1.0 ^b^	8.2 ± 0.1 ^c^	24.4 ± 1.0 ^c^
C-N	28.7 ± 2.7 ^b^	0 ± 0 ^d^	32.2 ± 0.1 ^a^	39.1 ± 2.8 ^a^
LF	4.6 ± 2.1 ^e^	48.5 ± 5.9 ^b^	8.3 ± 3.1 ^c^	38.6 ± 0.8 ^a^
BSA	30.5 ± 1.4 ^b^	35.3 ± 1.0 ^c^	6.2 ± 0.8 ^c^	28.0 ± 0.6 ^b^
CN	0 ± 0 ^f^	58.5 ± 0.3 ^a^	2.4 ± 0 ^d^	39.1 ± 0.3 ^a^
Nisin	100 ± 0 ^a^	0 ± 0 ^d^	0 ± 0 ^d^	0 ± 0 ^d^

Values in the same column with different superscript letters are statistically different (*p* < 0.05). L-N: lactoferrin–nisin nanoparticles, B-N: bovine serum albumin–nisin nanoparticles, C-N: casein–nisin nanoparticles.

**Table 2:** Minimum inhibitory concentration (MIC) and minimum bactericidal concentration (MBC) of the different samples.

Samples	MIC (mg/mL)			MBC (mg/mL)		
	*L. monocytogenes*	*S. aureus*	*B. cereus*	*L. monocytogenes*	*S. aureus*	*B. cereus*
L-N	0.625	0.625	1.25	1.25	1.25	1.25
B-N	0.625	0.625	1.25	1.25	1.25	1.25
C-N	1.25	2.5	2.5	2.5	2.5	/
Nisin	0.625	0.625	1.25	0.625	1.25	1.25

L-N: lactoferrin–nisin nanoparticles, B-N: bovine serum albumin–nisin nanoparticles, C-N: casein–nisin nanoparticles.

## Data Availability

The original contributions presented in the study are included in the article/Supplementary Materials, further inquiries can be directed to the corresponding author.

## Supplementary Materials

The following supporting information can be downloaded at: https://www.mdpi.com/article/10.3390/foods13111606/s1, Figure S1: Antibacterial effects of different samples on *L. monocytogenes* (left) and *S. aureus* (right); Table S1: Particle size, PDI, and zeta potential of different samples. References [77,78,79,80,81,82,83] are cited in the supplementary materials.
