# Supplementary material for: The Effect of Nanoscale Modification of Nisin by Different Milk-Derived Proteins on Its Physicochemical Properties and Antibacterial Activity
Source: Foods. 2024 May 22;13(11):1606. doi: 10.3390/foods13111606 (PMC11171616; doi:10.3390/foods13111606)
Supplement: Supplementary file 1 [file foods-13-01606-s001.zip › foods-2991541-supplementary.pdf]

The effect of nanoscale modification of nisin by different milk derived proteins on its physicochemical properties and antibacterial activity

Contents:

Supporting Information: Methods Characterization of different milk protein-nisin nanoparticles.

Supporting Information: Table S1. Particle size, PDI, and zeta potential of different samples.

Supporting Information: Figure S1. Antibacterial effects of different samples on *L. monocytogenes* (left) and *S. aureus* (right).

1.Characterization of different milk protein-nisin nanoparticles

1.1. Determination of particle size, polydispersity index (PDI), and zeta potential

The particle size, PDI, and zeta potential of milk proteins, nisin, and its nanoparticles were determined using a Zetasizer Nano ZS instrument (Malvern Instruments, Malvern, UK) (Wu et al., 2023). The test temperature was 25°C, the refractive index of water was 1.33, and the refractive index of samples were 1.45.

1.2. Observation of apparent morphology

The apparent morphology of nisin and milk protein-nisin nanoparticles was observed by scanning electron microscopy (SEM, S-4700, Hitachi, Tokyo, Japan) (Zhang et al., 2022). The freeze-dried samples were uniformly coated onto the

conductive adhesive and sprayed with gold. The observation conditions were 25°C, accelerating voltage was 10 kV, magnification was 5,000.

1.3. Determination of Fourier transform infrared (FTIR)

The molecular interaction forces between milk proteins and nisin were analyzed using FTIR spectroscopy (VERTEX 70, Bruker, Germany) (Zhang et al., 2022). The dried samples were thoroughly mixed with KBr in the ratio of 1:100 and ground evenly, pressed into tablets and subjected to FTIR spectroscopy. Spectra were collected over a range of 400-4000 cm^{-1} with a resolution of 4 cm^{-1} and an average number of scans of 32, with a baseline of pure KBr powder.

1.4. Determination of ultraviolet-visible (UV-vis) absorption

The UV-vis absorption spectra of milk proteins, nisin, and its nanoparticles were measured using a UV-vis spectrophotometer (UV-1800, Shimadzu, Japan) (Li et al., 2019). The test temperature was 25°C, the optical range of the quartz cuvette was 1 cm, scanning wavelength range was 190-350 nm with a scanning interval of 1 nm.

1.5. Determination of fluorescence spectra

The fluorescence emission spectra of milk proteins, nisin, and its nanoparticles were recorded using a fluorescence spectrometer (F-4600, Hitachi, Tokyo, Japan) (Li et al., 2019). The samples were diluted ten times before measurement. The test temperature was 25°C, the excitation wavelength was 280 nm, the scanning range was 300-500 nm, the scanning speed was 1200 nm/min, and all excitation and emission slits were fixed at 5.0 nm.

1.6. Determination of circular dichroism (CD)

The CD spectra of milk proteins, nisin, and its nanoparticles were recorded between 190-240 nm using a CD spectrometer (J-1500, JASCO, Japan), and the secondary structure (α-helix, β-sheet, β-turn, and random coil) content of the samples was calculated and analyzed (Liu et al., 2022). The test was performed at 25°C with a scanning speed of 100 nm/min, a sensitivity of 200 mdeg, and a cuvette optical range of 1 mm.

1.7. Determination of X-ray diffraction (XRD)

The crystalline properties of milk proteins, nisin, and its nanoparticles were determined using an X-ray diffractometer (D8 ADVANCE, Rigaku, Osaka, Japan) (Kang et al., 2023). Cu-Kα radiation was generated at a voltage of 40 kV and a current of 40 mA with a scanning angle ranging from 5° to 80° (2θ) and a scanning speed of 5°/min.

1.8. Determination of differential scanning calorimeter (DSC)

The thermodynamic properties of milk proteins, nisin, and its nanoparticles were determined using a DSC (204 F1, NETZSCH, Selb, Germany) (Yang et al., 2022). 5-10 mg of the sample was weighed into an aluminum pot and sealed, while an empty aluminum pot was used as a reference, and the temperature was varied over the range of 25-350°C under constant nitrogen purge with a temperature increase rate of 10°C/min.

Table S1 Particle size, PDI, and zeta potential of different samples

Samples	Size (nm)	PDI	Zeta potential (mV)
L-N	29.83 ± 2.42^{e}	0.38 ± 0.01^{c}	7.04 ± 0.31^{b}
B-N	69.86 ± 0.61^{d}	0.28 ± 0.00^{d}	-0.99 ± 0.23^{d}
C-N	140.50 ± 0.70^{c}	0.18 ± 0.02^{e}	10.84 ± 0.82^{a}
LF	15.91 ± 0.32^{f}	0.47 ± 0.02^{b}	12.17 ± 1.72^{a}
BSA	13.94 ± 0.23^{f}	0.70 ± 0.01^{a}	-14.2 ± 0.7^{e}
CN	145.43 ± 4.83^{b}	0.46 ± 0.10^{bc}	-27.57 ± 0.65^{f}
Nisin	150.37 ± 3.75^{a}	0.49 ± 0.08^{b}	4.44 ± 0.59^{c}

* Data were expressed as mean ± standard deviation (n = 3). The values in the same column with different superscript letters were statistically different ($p < 0.05$).

As shown in Table S1, the particle size of nisin and its nanoparticles were in the order of nisin > C-N > B-N > L-N from largest to smallest, which showed that all the nanoparticles were smaller than nisin, with the smallest particle size of L-N, with an average particle size of 29.83 ± 2.42 nm. Based on research, it appeared that when milk proteins bound with nisin, it could effectively decrease the particle size of nisin. This reduction was likely due to the use of ultrasound nanotechnology, which caused a cavitation effect and led to a decrease in the size of nanoparticles (Wang et al., 2023). PDI is mainly used to characterize the dispersion of the sample. The smaller the PDI, the more homogeneous the dispersion of the sample (Quichaba et al., 2023). As seen from Table S1, the PDI of the three nanoparticles was significantly smaller than those of the individual components, indicating improved dispersion of the nanoparticles.

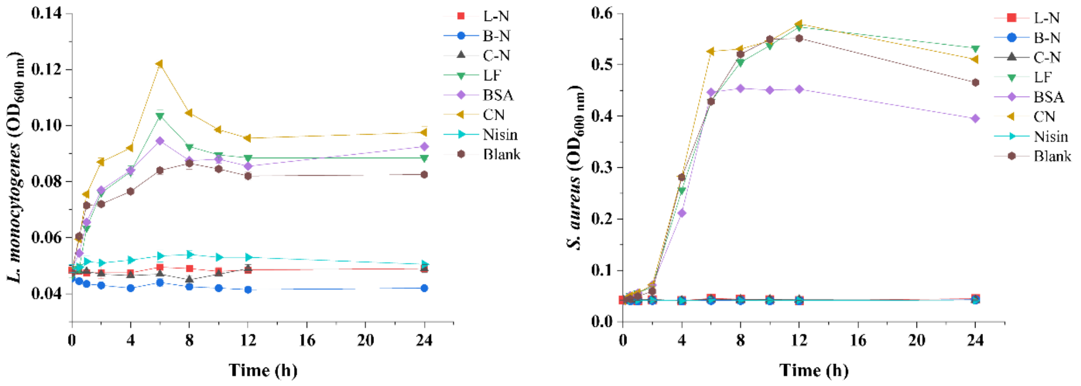

Figure S1 Antibacterial effects of different samples on *L. monocytogenes* (left) and *S. aureus* (right).

The result was shown in **Figure S1**, the initial OD_{600nm} of both the experimental and control groups for the proliferation of *L. monocytogenes* and *S. aureus* was relatively low. However, with prolonged incubation time, the OD_{600nm} of the three proteins and the blank control group gradually increased, indicating unhindered bacterial growth and an increase in bacterial quantity. Conversely, the OD_{600nm} of the three nanoparticles and the nisin group remained consistently low, suggesting inhibited bacterial growth. These results indicated that the three proteins did not exhibit antibacterial effects, whereas the nanoparticles and nisin demonstrated effective antibacterial activity.

Reference

Kang, L., Liang, Q., Chen, H., et al. (2023). Insights into ultrasonic treatment on the properties of pullulan/oat protein/nisin composite film: mechanical, structural and physicochemical properties. Food Chemistry, 402:134237.

Li, J., Pan, D., Yi, J., et al. (2019). Protective effect of β-cyclodextrin on stability of nisin and corresponding interactions involved. Carbohydrate Polymers, 223:115115.

Liu, X., Ibarra-Sanchez, L.A., Miller, M.J., et al. (2022). Fabrication of zein-modified starch nanoparticle complexes via microfluidic chip and encapsulation of nisin. Current Research in Food Science, 5:1110-1117.

Quichaba, M.B., Moya Moreira, T.F., de Oliveira, A., et al. (2023). Biopreservatives against foodborne bacteria: combined effect of nisin and nanoncapsulated curcumin and co-encapsulation of nisin and curcumin. Journal of Food Science and Technology-Mysore, 60(2):581-589.

Wang, Z., Zhao, H., Tao, H., et al. (2023). Ultrasound improves the physicochemical and foam properties of whey protein microgel. Frontiers in Nutrition, 10:1140737.

Wu, C., Zhi, Z., Duan, M., et al. (2023). Insights into the formation of carboxymethyl chitosan-nisin nanogels for sustainable antibacterial activity. Food Chemistry, 402:134260.

Yang, Z., Shen, C., Rao, J., et al. (2022). Biodegradable gelatin/pullulan aerogel modified by a green strategy: Characterization and antimicrobial activity. Food Packaging and Shelf Life, 34:100957.

Zhang, L., Chen, D., Yu, D., et al. (2022). Modulating physicochemical, antimicrobial and release properties of chitosan/zein bilayer films with curcumin/nisin-loaded pectin nanoparticles. Food Hydrocolloids, 133:107955.
